# The antimicrobial effect of *Limosilactobacillus reuteri* as probiotic on oral bacteria: A scoping review

**DOI:** 10.12688/f1000research.139697.1

**Published:** 2023-11-23

**Authors:** Nissia Ananda, Dewi Fatma Suniarti, Endang Winiati Bachtiar

**Affiliations:** 1 Dental Department, Universitas Indonesia Hospital, Universitas Indonesia, Depok, West Java, 16424, Indonesia; 2 Department of Oral Biology, Oral Science Research Center, Faculty of Dentistry, Universitas Indonesia, Jakarta, 10430, Indonesia

**Keywords:** antimicrobial, dentistry, Limosilactobacillus reuteri, Lactobacillus reuteri, oral bacteria, oral microbiome, oral probiotic

## Abstract

Dysbiosis among oral microbial community in the oral cavity can lead to several oral diseases. Probiotic therapy is known to correct these imbalances. *Limosilactobacillus reuteri* is one of the most studied strains of probiotics and can control oral microbiota through reuterin, a wide-spectrum antimicrobial agent. The objective of this review was to evaluate the effect of the antimicrobial activity of *Limosilactobacillus reuteri* on the oral bacteria of humans. This review used PubMed, Scopus, EMBASE, ScienceDirect, and Google Scholar databases as bibliographic resources. Studies with matching keywords were analyzed and screened with PRISMA-ScR recommendations. Sixteen articles were selected for this review, which included a total of 832 patients. Based on this review, *Limosilactobacillus reuteri* has a strong antibacterial effect against *Streptococcus mutans* in healthy individuals but is not effective against *Lactobacillus.* Additionally, it has a significant antibacterial effect against *Porphiromonas gingivalis* in patients with periodontitis, although its effectiveness is not stable in patients with peri-implant infections. Furthermore, *Limosilactobacillus reuteri*has varying results against other bacteria, indicating the need for further extensive research to ensure its efficacy.

## Introduction

Microbiome is the community of microbial residents in human body and oral microbiome is the microorganisms resided in the oral cavity. It is known that oral microbiome is the second largest microbial habitat in humans after the gut and oral bacteria are the main components of this oral microbiota. ^1^ It is a home for more than 700 bacterial species because oral cavity can provide a favorable habitat for the growth of microorganisms. ^1^^,^^2^ Bacteria could have a stable environment to survive in oral cavity with the constant average of temperature is 37°C and steady pH of 6.5-7. ^1^

Generally oral microbiome consists of a core microbiome and a variable microbiome. The core microbiome is community of predominant species that are present in various parts of the body under healthy condition. Meanwhile, a variable microbiome is a community of microorganisms species that varies and is exclusive between individuals because it has evolved in response to unique lifestyle and genotypic determinants. ^1^

The fetus inside the uterus is usually sterile. The baby comes in contact with the microflora for the first-time during delivery, specifically with the uterine microflora and the mother’s vaginal microflora. At birth, the baby then also comes into contact with atmospheric microorganisms. Normally, the newborn’s oral cavity is sterile and regularly inoculated with microorganisms from the first feeding onward, and the process of acquisition of resident oral microflora begins. ^1^ Previous studies suggest that there were significant changes in the composition of the microbiome over time in humans, and this was caused by many factors. One of the influencing factors is the decrease in salivary flow rate and oral pH as an individual gets older. ^3^

Oral microbiomes usually reside in the oral cavity as biofilms. Maintaining the homeostasis of this biofilm is important to protect the oral cavity. ^1^ The physiology and ecology of the microbiota are closely related to the host, this has an impact on the promotion of health or the development of disease. Since the oral cavity is the main gateway to the body, it can also lead to the spread of infection to other parts of the body causing systemic disease. ^4^ It is currently recognized that unresolved inflammation promotes conversion to a dysbiotic community, and host-related intrinsic factors and defense mechanisms may play a major role in disease pathogenesis. ^2^^,^^5^ Antibiotics are commonly used in dentistry to treat oral diseases, not infrequently they are also used as prophylactic treatment. ^6^ On the other hand, excessive use of antibiotics can increase the risk of developing antimicrobial resistance. ^7^ Unfortunately, the current condition of antimicrobial resistance has been declared by the WHO (World Health Organization) as a global public health emergency and is known as a silent pandemic. ^8^

Probiotics are living and viable microorganisms which in adequate quantities will provide benefits to the host. ^9^ Probiotics can improve oral flora through a bacteriotherapy mechanism which is increasing the composition of harmless microflora to maintain or restore beneficial oral flora and modulating the oral microbiota to prevent pathogen colonization. ^10^ In addition to bacteriotherapy mechanisms, probiotics can also prevent or treat oral microbiota dysbiosis through stimulation of the host’s immune system and produce molecules or substances with antimicrobial effects. ^9^^,^^11^^,^^12^ These concept provides an alternative therapy to fight infection with fewer side effects and is safer than antibiotics. ^10^

Over the past few years, probiotic therapy has been used as an adjunct treatment in clinical dentistry. ^13^ For oral or dental purposes, probiotics need to survive in the oral ecosystem. ^14^ In terms of oral health, *Limosilactobacillus reuteri* has been extensively researched and is considered one of the most studied probiotics. ^15^ *Limosilactobacillus reuteri* is known for its ability to control oral microbiota through reuterin, a wide-spectrum antimicrobial agent. ^16^ However, some studies discovered that the *Limosilactobacilluss reuteri* probiotic should not be recommended as an adjunct treatment for oral infection as there were no microbiota alterations between the test and control groups. ^11^^,^^17^^,^^18^ The purpose of this study is to provide a comprehensive overview of the existing literature on the antimicrobial effect of *Limosilactobacillus reuteri* on oral bacteria. This review aims to synthesize and map out prior research in order to identify knowledge gaps and research needs related to this topic. By examining the breadth and depth of available literature, this review will evaluate the quality and quantity of existing research on the subject, and inform future research and practice. Ultimately, this scoping review aims to provide a foundation for future research and practice in this important area of alternative antimicrobial drug research.

### Objective

This review evaluated the effect of the antimicrobial activity of *Limosilactobacillus reuteri* on the oral microbiota of humans.

## Methods

### Scoping review methods

To achieve this study’s objective, the Preferred Reporting Items for Systematic Reviews and Meta-Analyses-Extension for Scoping Reviews (PRISMA-ScR) were applied.

### Research questions

What is known from the literature about the antibacterial effect of *Limosilactobacillus reuteri* on oral bacteria in humans?

### Search methods

We ran a search for full-text manuscripts written in English in several databases, such as PubMed, Scopus, EMBASE, ScienceDirect, and Google Scholar. There were no limitations regarding the articles’ publishing date at the search stage. The search was performed by identifying studies with the terms “( *Lactobacillus reuteri* OR *Limosilactobacillus reuteri* OR Probiotic) AND (Antimicrobial OR Antibacterial) AND (Oral OR Dental)”. Google Scholar was included in our search strategy to ensure a comprehensive search. However, due to the large number of results obtained from Google Scholar, we limited our screening to the first 10 pages of results, which were sorted by relevance.

### Selection of studies

Rayyan *,* developed and manufactured by Qatar Computing Research Institute in Doha, Qatar, was used as a tool for the screening and selection of studies for inclusion in this study. We imported the search results into Rayyan, de-duplicated them, and then two reviewers (NA and DFS) screened the title and abstract of each study to determine its relevance to the research question. Studies that were deemed potentially relevant by either reviewer were included for full-text review. During the full-text review stage, each reviewer independently assessed the eligibility of each study based on predetermined inclusion and exclusion criteria. Any disagreements were resolved through discussion or by involving a third reviewer (EWB). Finally, the studies that met the inclusion criteria were selected for data extraction and analysis. Rayyan was used to record the results of the screening and selection process, including the number of studies included and excluded at each stage, and to facilitate the creation of a Preferred Reporting Items for Systematic Reviews and Meta-Analyses (PRISMA) flow diagram.

### Eligibility criteria

The inclusion criteria for the studies in this review were based on PICOS.

i. Population, or participants and conditions of interest: Oral or Dental bacteria in humans
ii. Interventions or exposures: Exposure to *Limosilactobacilluss reuteri*
iii. Comparisons or control groups: a control group of microbiomes that was not exposed *to Limosilactobacillus Reuteri*
iv. Outcomes of interest: Quantity of Oral Bacteria (Antimicrobial effect)
v. Study designs: Randomized controlled trials (RCTs) and non-RCTs, case-control studies, cross-sectional, as well as prospective and retrospective cohort studies were considered for inclusion. Systematic review studies were also included to explore the studies in the review that are relevant to this study.

The exclusion criteria for this review were letters to editors, commentaries, case reports, articles with non-human samples, and non-oral/dental research articles.

### Data extraction

Data were extracted from each study based on the author, year, country, study design, subject criteria, number of samples, groups in the research, strain of *Limosilactobacillus reuteri* used, delivery systems, other accompanying treatment, duration of intervention, bacteria affected, and the statistical analysis of oral bacteria count.

### Methodological quality assesment

We used version 2 of the Cochrane Risk-Of-Bias (RoB 2) Tool, developed and manufactured by Cochrane Collaboration, a non-profit organization based in London, United Kingdom, to assess the methodological quality of the studies. The risk of bias in each individual study was assessed by two reviewers, independently. The criteria of this assessment consisted of the following five domains: the randomisation process, deviations from intended interventions, missing outcome data, measurement of the outcome, and selection of the reported result.

## Results

### Search results

**Figure 1.**
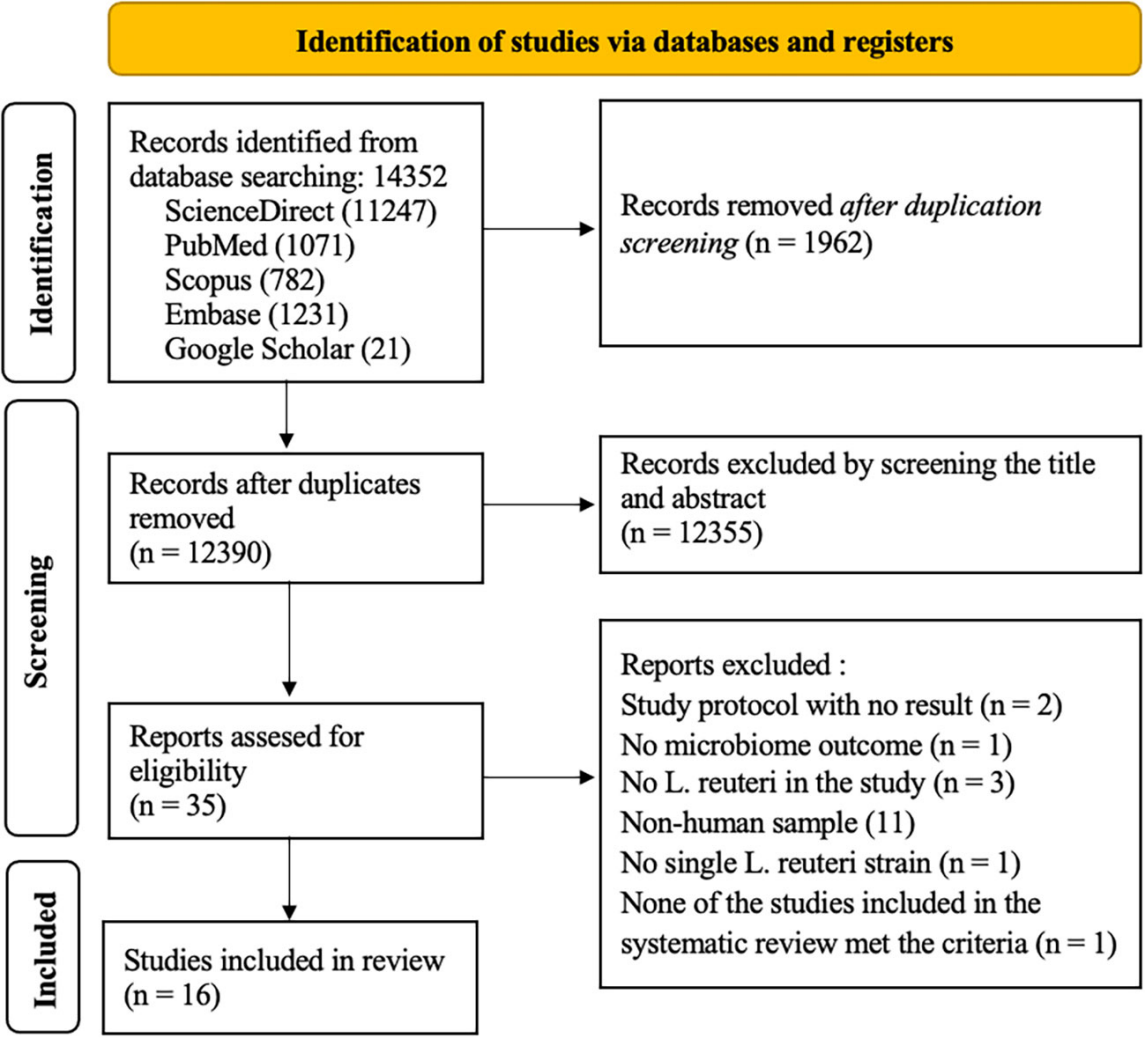
Flowchart of the scoping review.

The electronic literature research process was conducted in April 2023, and 14352 studies were identified. The studies were obtained from the following four databases: PubMed, Scopus, EMBASE, ScienceDirect, and Google Scholar. A total of 1962 studies were eliminated due to duplication, 12355 studies were excluded by title and abstract screening. From the remaining thirty-five studies, nineteen studies were also excluded due to the following reasons: their subject was not human, there was no microbiome outcome in the study, the studies in the systematic review did not met the criteria, the study was only study protocol with no result, and the probiotic used was not *Limosilactobacillus reuteri* or *Limosilactobacillus reuteri* mixed with other bacteria. Therefore, sixteen studies were selected in this review for analysis ( Table 1). The PRISMA flowchart of this systematic review is presented in Figure 1.

**Table 1.** Included studies.

Author (Year)	Country	Design of study	Subject	Patients, *n*	Groups, *n*	Strain of L. Reuteri	Delivery systems	Accompanied with other treatment	Duration of intervention	Observation Period	Bacteria affected	Main finding
Caglar *et al.* (2008)	Turkey	RCT	Healthy young women aged 20 years old	20	G1: exposed to L. reuteri (n=10) G2: control group (n=10)	*L. reuteri* ATCC 55730/ *L. reuteri* ATCC PTA 5289 (1.1 x 10 ^8^ CFU)	Medical device contains a pouch in which the probiotic or placebo lozenge can be inserted in	No	10 days, with 15 minutes of intervention/day	10 days	*Salivary mutans streptococci* *Salivary lactobacilli*	G1 exhibit significantly reduced number of *Salivary mutans streptococci* on day 10 *P* = 0.016 No statistically significant difference between G1 and G2 on *Salivary lactobacilli* count on day 10
Galofre *et al.* (2017)	Spain	RCT	Patients that partially edentulous and had implants with mucositis or peri-implantitis	44	G1: mucositis patients (n=22) G1.1: mucositis patients who received probiotic lozenge (n=11) G1.2: mucositis patients who received placebo lozenge (n=11) G2: peri-implantitis patients (n=22) G2.1: peri-implantitis patients who received probiotic lozenge (n=11) G2.2: peri-implantitis patients who received placebo lozenge (n=11)	*L. reuteri* (1 × 10 ^8^ living cells of DSM 17938 and 1 × 10 ^8^ living cells of ATCC PTA 5289)	Lozenge	Supragingival prophylaxis in the mucositis implants Subgingival non-surgical mechanical therapy in peri-implantitis implants and titanium curettes	every day for 30 days	30 days 60 days 90 days	*Aggregatibacter actinomycetemcomitans* *Tannerella forsythia* *Porphyromonas gingivalis* *Treponema denticola* *Prevotella intermedia* *Peptostreptococcus micros* *Fusobacterium nucleatum* *Campylobacter rectus* *Eikenella corrodens*	Significantly reduced *Porphyromonas gingivalis* between G1.1 and G1.2 (bacterial count on day 90 minus bacterial count on baseline) P = 0.031 However no statistically significant difference between G1.1 and G1.2, also G.21 and G2.2 in other peri-implant microbiota
Caglar *et al.* (2007)	Turkey	RCT	Healthy young adults, 21-24 years of age	80 (44 women, 36 men)	G1: consumed one chewing gum with probiotic bacteria three times daily (n=20) G2: consumed two chewing gums with xylitol three times daily (n=20) G3: consumed two chewing gums with probiotic bacteria and four with xylitol daily (n=20) G4: chewed placebo gums without active ingredients three times a day (n=20)	*L. reuteri* (ATCC 55730 at a dose of 1×108 CFU/gum and ATCC PTA 5289 at a dose of 1× 108 CFU/gum)	Lozenge	Brush the teeth twice a day with fluoride-containing toothpaste	3 weeks	3 weeks	*Salivary mutans streptococci* *Salivary lactobacilli*	G1 and G2 exhibit significantly reduced number of *Salivary mutans streptococci* on week 3 compared to baseline (P < 0.05) G3 and G4 showed no statistically significant difference *Salivary mutans streptococci* count between baseline and week 3 G1, G2, G3, G4 showed no significant difference S *alivary lactobacilli* scores between baseline and week 3
Pena *et al.* (2018)	Spain	RCT	Mucositis patients with dental implants	50	G1: Probiotic group (given probiotic tablet on day 15-45) n = 25 G2: Placebo group (given placebo tablet on day 15-45) n = 25	*L. reuteri (*DSM 17938 and ATCC PTA 5289)	Tablet	Mechanical debridement of dental implant with adjunctive administration of a 0.12% chlorhexidine mouthrinse (day 0-15)	intervention on day 15-45	day - 15 day - 45 day - 135	*Aggregatibacter actinomycetemcomitans (Aa)* *Tannerella forsythia (Tf)* *Porphyromonas gingivalis (Pg)* *Treponema denticola (Td)* *Prevotella intermedia (Pi)* *Peptostreptococcus micros (Pm)* *Fusobacterium nucleatum (Fn)* *Campylobacter rectus (Cr)* *Eikenella corrodens (Ec)*	Significantly reduced *Peptostreptococcus micros* in G1 compared to G2 (bacterial count on day 45 minus bacterial count on day 15) P = 0.001 Significantly reduced *Peptostreptococcus micros* and *Fusobacterium nucleatum* in G1 compared to G2 (bacterial count on day 135 minus bacterial count on baseline) P (Pm) = 0.045 P (Fn) = 0.034 However no statistically significant difference between G1 and G2 in other peri-implant microbiota
Nikawa *et al.* (2004)	Japan	RCT	Healthy female subjects who were 20 years old	40	G1: given placebo yoghurt daily for 2 weeks, and then given reuteri yoghurt daily for another 2 weeks n = 20 G2: given reuteri yoghurt daily for the first 2 weeks, then given placebo yoghurt daily for the next 2 weeks. n = 20	*L. reuteri* SD2112 (ATCC55730)	Yoghurt	-	2 weeks of intervention	4 weeks	*Oral mutans streptococci*	*L. reuteri* significantly reduced the *oral mutans streptococci* in each group (P<0.05)
Hallstrom *et al.* (2015)	Sweden	RCT	Patients with peri-implant mucositis	46	G1: test group (the in-office treatment included topical application of oil containing L. reuteri around selected implant and patient given probiotic tablet twice daily for 3 months) n = 22 G2: control group (got the same in-office mechanical treatment with topical application of placebo oil and given placebo tablet) n = 24	*L. reuteri* strains DSM 17938 and ATCC PTA 5289	Topical oil and tablet	titanium curretes, polishing using a rubber cup and polishing paste	3 months	week 0 week 4 week 12 week 26	*Porphyromonas gingivalis* (FDC381) *Prevotella intermedia* (ATCC26511) *Prevotella nigrescens* (ATCC 33563) *Tannerella forsythia* (ATCC43037) *Aggregatibacter actinomycetemcomitans* (FDC Y4) *Fusobacterium nucleatum* (ATCC10953) *Treponema Denticola* (OMGS3271) *Parvimonas micra* (OMGS2852) *Campylobacter rectus* (ATCC33238) *Porphymonas endodontis* (OMGS1205) *Filifactor alocis* (ATCC35896) *Prevotella tannerae* (ATCC51259)	No major alterations over time or differences between G1 and G2 were recorded
Lauritano *et al.* (2018)	Italy	RCT	Patients with peri-implant mucositis	10	G1: consumed one chewing gum with probiotic bacteria one per day (n=5) G2: chewed placebo tablet one per day (n=5)	*L.reuteri* DSM 17938 and ATCC PTA 5289	Chewing tablet	non-surgical mechanical therapy	28 days	28 days	*Porphyromonas gingivalis* *Tannerella forsythia* *Treponema denticola*	No statistically significant difference between G1 and G2 in other peri-implant microbiota
Caglar *et al.* (2006)	Turkey	RCT	Healthy young adults, 21-24 years of age	120 (71 male, 49 female)	G1: drank 200 ml water through a prepared straw containing probiotic bacteria once daily G2: drank 200 ml water through a placebo straw without bacteria once daily G3: ingested one sucking tablet with probiotic bacteria once daily G4: ingested one sucking tablet without bacteria once daily	Probiotic straw consisted of a telescopic polypropene membrane with an oil droplet containing *L. reuteri* ATCC 55730 (minimum 10 ^8^ CFU/straw) attached to its inner part Tablet consisted of *L. reuteri* ATCC 55730 (10 ^8^ CFU/tablet)	Straw Tablet	-	3 weeks	3 weeks	*Salivary mutans streptococci* *Salivary lactobacilli*	G1 and G3 showed significantly reduced number of *mutans streptococci* between baseline and end of intervention G2 and G4 showed no statistically differences of *mutans streptococci* score on baseline and on the 3rd week G1, G2, G3, and G4 showed no statistically differences of *Lactobacilli score* on baseline and on the 3rd week
Tada *et al.* (2017)	Japan	RCT	Patients with peri-implantitis	30	G1: exposed to L. reuteri (n=15) G2: control group (n=15)	One probiotics tablet contained 1x108 CFU *L. reuteri* strains DSM 17938 and ATCC PTA 5289	Tablet	supragingival scaling, prescribtion of azythromycin 500 mg/day for 3 days	24 weeks	24 weeks	*Fusobacterium nucleatum* *Porphyromonas gingivalis* *Prevotella intermedia* *Aggregatibacter actinomycetemcomitans* *Treponema denticola* *Tannerella forsythia*	No statistically significant difference between G1 and G2 in other peri-implant microbiota
Laleman *et al.* (2019)	Belgium	RCT	Patients with peri-implantitis	19	G1: topical application of the probiotic drops after the treatment and consume probiotic lozenges G2: topical application of the placebo drops after the treatment and consume placebo lozenges	*L. reuteri* DSM 17938 and *L. reuteri* ATCC PTA 5289	Drop	Full mouth prophylaxis Mechanical debridement of the peri-implant sites Peri-implant pockets were subgingivally air polished	12 weeks	baseline week 6 week 12 week 24	*Porphyromonas gingivalis* *Prevotella intermedia* *Fusobacterium nucleatum* *Aggregatibacter actinomycetemcomitans*	Significantly reduced *Porphyromonas gingivalis* in G1 compared to G2 on week 6 observation in the saliva sample P = 0.006 Significantly reduced *Aggregatibacter actinomycetemcomitans* in G1 compared to G2 on week 12 and week 24 observations in the saliva sample, and similar result on the week 24 observation in the tongue sample P (week12-saliva) = 0.034 P (week 24-saliva) = 0.040 P (week 24-tongue) < 0.001 Significantly reduced *Prevotella intermedia* in G1 compared to G2 on week 6, week 12 and week 24 observations in the tongue sample, and similar result on the week 24 observation in the subgingival sample p < 0.001
Alamoudi *et al.* (2018)	Kingdom of Saudia Arabia	RCT	Healthy children aged 3-6 years	178	G1: received *L. reuteri* probiotic lozenges (n = 90) G2: received placebo lozenges (n = 88)	*L. reuteri* DSM 17938 and *L. reuteri* ATCC PTA 5289	Lozenge	Regular dental hygiene for the children using a “pea-size” amount of provided toothpaste with 500 ppm fluoride twice daily.	28 days	28 days	*Mutans streptococci* *Lactobacillus*	G1 showed significantly lower microbial counts compared to G2 for both salivary *Mutans streptococci* (P=0.000) and *Lactobacilli* (P=0.020)
Elsadek *et al.* (2020)	Saudi Arabia	Non-RCT experimental	Periodontitis patients with Diabetes Mellitus	60	G1: administered probiotic and root surface debridement G2: rendered with root surface debridement alone	*L. reuteri* (DSM 17938 and ATCC PTA 5289) at 2 x 10 ^8^ CFU	Tablet	Root surface debridement	Two tablets/day for 3 weeks	Basline 3 months	*Porphyromonas gingivalis* *Tannerella forsythia* *Treponema denticola*	G1 exhibit significantly reduced number of *Porphyromonas gingivalis, Tannerella forsythia,* and *Treponema denticola* compared to G2 on 3 months evaluation
Vivekananda *et al.* (2010)	India	RCT with split mouth design	Chronic periodontitis patients	30	G1: SRP (Scaling root planing) treated quadrants + probiotic lozenge G2: SRP treated quadrants + placebo lozenge G3: Non-SRP quadrants + probiotic lozenge G4: Non-SRP quadrants + placebo lozenge	*L. reuteri* DSM 17938 (1 x 10 ^8^ CFU) and *L. reuteri* ATCC PTA 5289 (1 x 10 ^8^ CFU)	Lozenge	Scaling root planing (G1 and G2)	Day 21 to day 42 (21 days)	Baseline Day – 21 Day – 42	*Aggregatibacter actinomycetemcomitans* *Porphyromonas gingivalis* *Prevotella intermedia*	G1 and G3 were able to significantly reduce the CFU counts of the *Aggregatibacter actinomycetemcomitans, Porphyromonas gingivalis, and Prevotella intermedia*
Badri *et al.* (2020)	Makkah, Saudi Arabia	RCT	Healthy children aged 8-12 years	53	G1: One lozenge of L. reuteri every day (n=18) G2: Chlorhexidine mouthwash everyday (n=17) G3: Control group (n=18)	*L. reuteri* DSM 17938 and *L. reuteri* ATCC PTA 5289	Lozenge	Brushing teeth	1 month	Day – 15 Day – 30	*S. mutans*	Significantly difference *S. mutans* count on day 15 and day 30: G3 > G1 > G2
Iniesta *et al.* (2012)	Spain	RCT	Patients with gingivitis	40	G1: exposed to *L. reuteri* (n=20) G2: control group (n=20)	Two strains of *L. reuteri* (DSM-17938 and ATCC PTA 5289) at a dose of 2 x 10 ^8^ CFU/tablet	Tablet	After screening, participants received tooth-polishing (rubber cup and abrasive paste) and provided with a new toothbrush, fluoride toothpaste and dental floss	28 days (baseline to week 4)	Baseline Week 4 Week 8	*Lactobacillus spp.* *Porphyromonas gingivalis* *Prevotella intermedia* *Fusobacterium nucleatum* *Parvimonas micra* *Campylobacter rectus* *Capnocytophaga* *Eikenella corrodens* *Tannerella forsythia* *Aggregatibacter actinomycetemcomitans*	In saliva: G1 showed significant reductions in total anaerobic counts after 4 weeks (p = 0.021) and counts of *Prevotella intermedia* after 8 weeks (p = 0.030) In subgingival: G1 showed significant reductions in the changes baseline to 4 weeks for *Porphyromonas gingivalis* counts (p = 0.008)
El-bagoory *et al.* (2021)	USA	RCT	Chronic periodontitis patients	12	G1: exposed to *L. reuteri* (n=6) G2: control group (n=6)	*L. reuteri* DSM 17938 (1X10 ^8^ CFU)	Suspension through blunt syringe delivered to subgingival sites	Scaling root planing	Four times of application: - Baseline - 1 week - 2 week - 4 week	Baseline 3 months 6 months	*Porphyromonas gingivalis*	G1 showed significantly reduced number of *Porphyromonas gingivalis* on the 3 months and 6 months evaluation

### General characteristics of included studies

Fifteen of the selected studies were randomized controlled trials (RCTs), and one study was a non-RCT experimental study. These studies were published between 2004 and 2021. Of the sixteen studies, six articles utilized healthy human subjects (four articles used healthy young adult humans, and two articles used healthy children). Three studies included in this study were conducted with peri-implant mucositis patients, two studies used peri-implantitis patients, and one study used both peri-implantitis and peri-implant mucositis patients. Additionally, one study was conducted with gingivitis patients, two studies used periodontitis patients, and one study used periodontitis patients with Diabetes Mellitus condition. A total of 832 patients were included in these studies. The duration of intervention in the included studies ranged from 10 days to 24 weeks. On the other hand, the observation periods in these studies also ranged from baseline to 26 weeks.

### Strains of *Limosilactobacillus reuteri* Used in Studies

In two studies originating from Turkey, a combination *of Limosilactobacillus reute*ri ATCC 55730 and ATCC PTA 5289 strains were used, while another two studies used only *the Limosilactobacillus reuteri* ATCC 55730 strain. Meanwhile, one study used only the *Limosilactobacillus reuteri* DSM 17938 strain, and eleven other studies used a combination *of Limosilactobacillus reut*eri DSM 17938 and ATCC PTA 5289 strains.

### Results of the quality assessment

**Figure 2.**
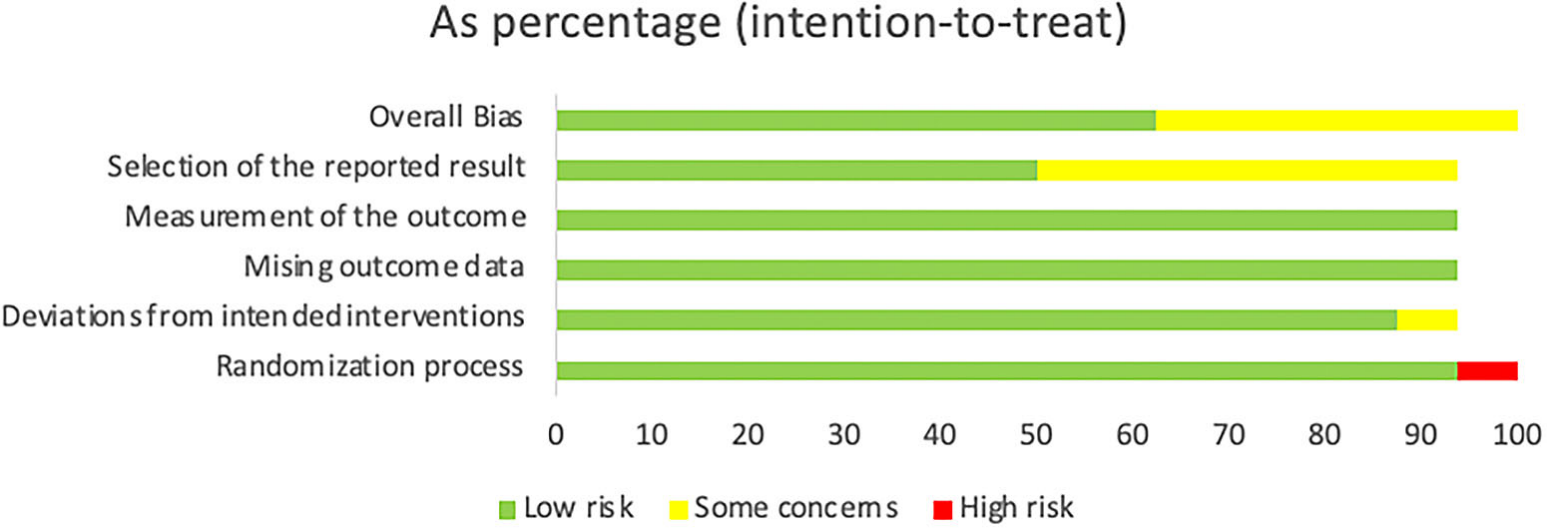
The distribution of risk-of-bias judgments within each bias domain.

During the quality assessment, it was found that the “low” percentage dominated the included articles, although there were still a few articles with the conclusion of “some concerns”. Additionally, only one article had a “high” result, and this article was a non-RCT experimental study. Therefore, it was reasonable to have a high bias value during this assessment (as shown in Figure 2 and Table 2). As a result, we concluded that all the studies included in this review are of good quality based on the Cochrane Risk-Of-Bias (RoB 2) Tool.

**Table 2.** Quality assesment of each studies.

Author	Risk of bias domain					
	Randomization process	Deviations from the intended interventions	Missing outcome data	Measurement of the outcome	Selection of the reported result	Overall Bias
Caglar *et al.* (2008)	Low	Some concerns	Low	Low	Some concerns	**Some concerns**
Galofre *et al.* (2017)	Low	Low	Low	Low	Low	**Low**
Caglar *et al.* (2007)	Low	Low	Low	Low	Low	**Low**
Pena *et al.* (2018)	Low	Low	Low	Low	Some concerns	**Some concerns**
Nikawa *et al.* (2004)	Low	Low	Low	Low	Some concerns	**Some concerns**
Hallstrom *et al.* (2015)	Low	Low	Low	Low	Some concerns	**Low**
Lauritano *et al.* (2018)	Low	Low	Low	Low	Low	**Low**
Caglar *et al.* (2006)	Low	Low	Low	Low	Some concerns	**Some concerns**
Tada *et al.* (2017)	Low	Low	Low	Low	Low	**Low**
Laleman *et al.* (2019)	Low	Low	Low	Low	Some concerns	**Some concerns**
Alamoudi *et al.* (2018)	Low	Low	Low	Low	Low	**Low**
Elsadek *et al.* (2020)	High	Low	Low	Low	Low	**High**
Vivekananda *et al.* (2010)	Low	Low	Low	Low	Some concerns	**Some concerns**
Badri *et al.* (2020)	Low	Low	Low	Low	Low	**Low**
Iniesta *et al.* (2012)	Low	Low	Low	Low	Low	**Low**
El-bagoory *et al.* (2021)	Low	Low	Low	Low	Low	**Low**

## Discussions

Antibiotic resistance is progressively increasing. This emphasizes the urgency of finding alternative antibacterial substances. ^19^ Many studies have tried to find alternative methods, other than antibiotics, to treat oral cavity diseases, one of which is through biofilm inhibitors. Quorum sensing (QS) research is one of the new frontiers in this regard. ^20^ Quorum sensing is a chemical signaling process of microorganisms that aims to monitor and regulate their population density. This chemical signaling system allows bacteria to coordinate their behavior and enhance their survival skills to respond to environmental changes through the growth and synthesis of biofilm matrix, enabling them to increase their resistance to conventional therapies such as antibiotics and antiseptics. ^20^^,^^21^

Generally, oral biofilms consist primarily of commensal species that are harmless to the host, but this balance may be altered by local or systemic causes, leading to a dysbiotic state or a condition characterized by increased number of pathogenic species. The involvement of quorum sensing mechanisms in facilitating biofilm formation helps bacteria perpetuate this dysbiotic state. ^20^ On the other side, interest in probiotics has tremendously risen over the last 10 years, and several health claims have been made regarding probiotics’ antibacterial activity. ^19^^,^^21^ In digestive studies, probiotics have a good adhesion to the gut, effectively enhancing intestinal epithelial homeostasis and interfering with the quorum sensing that favors a dysbiotic state. ^22^ Since probiotics act on biofilm composition, this current review is important as a preliminary knowledge to identify the effect of Lactobacillus reuteri on pathogenic bacteria in the oral cavity. ^15^

Six studies in this review analyzed the antibacterial properties of *Limosilactobacillus reuteri* against *Mutans streptococci* in healthy humans. Despite variations in probiotic delivery mechanisms, intervention duration, and observation periods, these studies found an overall positive antibacterial effect. Additionally, among the six studies, four used *Limosilactobacillus reuteri* ATCC 55730, and two studies combined this strain with *Limosilactobacillus reuteri* ATCC PTA 5289. ^23^^–^^27^ Two study performed by Alamoudi *et al.* (2018) and Badri *et al.* (2020) also used *Limosilactobacillus reuteri* DSM 17938 and ATCC PTA 5289. ^27^^,^^28^ Nikawa *et al.* (2004) conducted a study with a 2-week intervention period where *Limosilactobacillus reuteri* was administered via yoghurt, and the count of *Mutans streptococci* was observed for 4 weeks. ^26^ Five other studies observed the count of *Mutans streptococci* immediately after the intervention duration. ^23^^–^^25^^,^^28^ The results of these studies showed that *Limosilactobacillus reuteri* has an immediate and long-term effect of up to two weeks against *Mutans streptococci.* Related to this result, Caglar *et al.* (2009) conducted a study to investigate whether *Limosilactobacillus reuteri* ATCC 55730 survived in the oral cavity after the discontinuation of intervention. They found that *Limosilactobacillus reuteri* decreased gradually, where after 1 week, only 8% of bacteria were detected, and after 5 weeks, the bacteria were completely undetected. ^29^

One limitation of the findings on *Mutans streptococci* presented in this scoping review is that the subjects included in the literature are all healthy individuals. While these studies provide valuable insights into the prevalence and distribution of Streptococcus mutans in healthy populations, they may not necessarily reflect the same patterns observed in individuals with other oral health conditions. In fact, other studies have shown that there are significant differences in the levels of *Mutans streptococci* in individuals with severe periodontitis compared to healthy individuals. ^30^ Therefore, future research should aim to explore the distribution of *Mutans streptococci* in a more diverse range of populations, including those with varying levels of oral health conditions. This will help to better understand the role of *Mutans streptococci* in the development and progression of oral diseases and inform more targeted prevention and treatment strategies.

Four of the six previously mentioned studies also analyzed *salivary lactobacilli* count as a dependent variable. All of the studies performed with healthy adults had the result that *Limosilactobacillus reuteri* has no significant antibacterial effect on *salivary lactobacilli.* ^23^^–^^26^ However, a study by Alamoudi *et al.* (2018) with healthy children as participants, discovered a positive antibacterial effect of *Limosilactobacillus reuteri* on *salivary lactobacilli.* ^27^ This result may be influenced by the dental hygiene performed by the children using the provided fluoride toothpaste. It may also be due to the absence of *salivary lactobacilli* in newborns, and 40% of the 3-year-old population have it to varying degrees. Whereas *salivary lactobacilli* in adults depend on ecological conditions, such as pits and fissures or partially erupted wisdom teeth which provide an environment that supports its growth. ^31^

The studies performed on healthy subjects concerning *Mutans streptococci* and *salivary lactobacilli* count were based on the prior knowledge that both microbiomes are related to dental caries. One of the salivary *lactobacilli* species, *Lactobacillus acidophilus*, has characteristics that will increase significantly 2-3 months before dental caries appear. This phenomenon is called the “explosion of *Lactobacillus*”. However, the main oral acid production was not from *lactobacilli,* thereby making it only a secondary microbiome in dental caries. *Lactobacillus* cannot adhere to tooth surfaces on their own, as they need retention niches. On the other hand, *Streptococcus mutans* could produce extracellular polysaccharides to help them adhere to the tooth structure. *Streptococcus mutans’* main feature is its acidophilic nature. This allows it to become the dominant bacteria in the oral cavity during acidic conditions. Moreover, the intracellular polysaccharides in *Streptococcus mutans* create energy reserves, so the level of acid produced, especially lactic acid, remains constant even when external sugar intake is low. Hence, *Mutans streptococci* and salivary *lactobacilli* are strongly associated with dental caries. ^32^

Laleman *et al.* (2019), Tada *et al.* (2017, and Galofre *et al.* (2017) used peri-implantitis patients as their research subjects. These three studies analyzed the antibacterial effect of *Limosilactobacillus reuteri* DSM 17938 and ATCC PTA 5289 on *Porphyromonas gingivalis, Prevotella intermedia, Fusobacterium nucleatum, Aggregatibacter actinomycetemcomitans.* Furthermore, Tada *et al.* (2019) and Galofre *et al.* (2017) also analyzed *Treponema denticola and Tannerella forsythia,* whereas only Galofre *et al.* (2017) analyzed *Peptostreptococcus micros, Campylobacter rectus,* and *Eikenella corrodens.* All of the patients received non-surgical mechanical debridement of the peri-implant sites. ^18^^,^^33^^,^^34^ Two of these studies found that there were no significant differences between the treated and control groups in microbiome counts after 24 weeks and 30 days of intervention with *Limosilactobacillus reuteri* lozenges. ^18^^,^^34^ However, the study conducted by Laleman *et al.* (2019) that used probiotic drops as the drug delivery mechanism found different results compared to the other two studies. The intervention period was 12 weeks and the observation time was the baseline, week 6, week 12, and week 24. This study also evaluated the microbiome counts in the following three sites: saliva, tongue, and subgingival. The count of *Porphyromonas gingivalis* was only reduced in the saliva of the treated group at the week 6 observation. The count of *Aggregatibacter actinomycetemcomitans* significantly decreased in the saliva and tongue at week 12. On week 24, the count of *Aggregatibacter actinomycetemcomitans* also decreased in the saliva. Next, the count of *Prevotella intermedia* in the tongue of the treated group showed a significant decrease in all of the observation periods, and a decrease was also found in the subgingival area on week 24. ^33^ These varying results may be influenced by the drug delivery mechanisms and the sites evaluated. Nevertheless, we can conclude that the number of peri-implant microbiota evaluated at the subgingival site was generally not affected by *Limosilactobacillus reuteri,* whether administered by lozenge or drops. Thus, similar studies need to be conducted for the tongue area and saliva evaluation, and a larger number of samples needs to be used to obtain conclusive results.

Regarding peri-implant mucositis patients, four of the selected studies analyzed the antibacterial effect of *Limosilactobacillus reuteri* DSM 17938 and ATCC PTA 5289 on *Porphyromonas gingivalis, Tannerella forsythia,* and *Treponema denticola.* ^11^^,^^34^^–^^36^ Three of the previous four studies also analyzed *Prevotella intermedia, Aggregatibacter actinomycetemcomitans, Fusobacterium nucleatum,* and *Campylobacter rectus.* Two of these three studies analyzed *Peptostreptococcus micros* and *Eikenella corrodens,* whereas the other study analyzed *Fillifactor alocis, Porphyromonas endodontis,* and *Parvimonas micra.* ^34^^–^^36^ Overall, it can be concluded that *Limosilactobacillus reuteri* had no antibacterial effect on *Tannerella forsythia* and *Treponema denticola* in peri-implant mucositis patients as there was no positive result from the four studies despite differences in the intervention period, observation time, and drug delivery mechanisms. ^11^^,^^34^^–^^36^ Similar negative results from the three studies were also noted in *Prevotella intermedia, Aggregatibacter actinomycetemcomitans,* and *Campylobacter rectus.* Additionally, *Eikenella corrodens* was found to be not affected by *Lactobacillus reuteri* in two studies. ^34^^–^^36^

*Porphyromonas gingivalis* in peri-implant mucositis patients was found to be significantly affected by *Limosilactobacillus reuteri* application via probiotic lozenge for thirty days. However, this result was only positive on day-90 observation time while the study also performed observation on days 30 and 60. Nevertheless, as previously described, *Porphyromonas gingivalis* in the saliva of peri-implantitis patients was significantly affected by *Limosilactobacillus reuteri* in the week-6 of observation and there was no significant difference in the week 12 and week 24 samples. However, these results were considered inconclusive due to the small number of samples and require further research. ^34^

Pena *et al.* (2018) conducted a study with no statistically significant different results between the treated and control group in all peri-implant microbiota after administering *Limosilactobacillus reuteri* tablets from day 15 until day 45 of the study. However, he also analyzed the difference between the bacteria count on the day of observation and at the beginning of the study in each group, then he compared those numbers between both groups. This method resulted in significantly reduced *Peptostreptococcus micros* in the treated group compared to the control group, as indicated by the difference in bacterial counts between day 15 and day 45, and between day 135 and baseline. Another positive result was also found in *Fusobacterium nucleatum* in bacterial count on day 135 minus the bacterial count on the baseline. ^36^

One study conducted by Iniesta *et al.* (2012) provided evidence that the administration of *Lactobacillus reuteri* DSM 17938 and PTA 5289 probiotic tablets for 28 days effectively reduced the total anaerobic bacteria counts in saliva samples after 4 weeks and *Prevotella intermedia* counts after 8 weeks in gingivitis patients. This probiotic combination exhibited inhibitory effects against various periodontopathogens, such as *Porphyromonas gingivalis, Prevotella intermedia, Fusobacterium nucleatum, Parvimonas micra, Campylobacter rectus, Capno, Eikenella corrodens, Tannerella forsythia, and Aggregatibacter actinomycetemcomitans* in saliva samples. However, despite the significant total microbiological changes observed, these reductions in the target species did not translate into any clinically significant outcomes, as the inter-group differences in the clinical variables were not significant. This lack of clinical efficacy could be attributed to either the sample population or the short evaluation period. On the other hand, a significant reduction was observed only in *Porphyromonas gingivalis* counts in the subgingival sample from baseline to 4 weeks. These findings suggest that using *Lactobacillus reuteri* DSM 17938 and PTA 5289 probiotic tablets as an adjunct therapy could be promising for managing gingivitis and its associated periodontal pathogens, particularly *Porphyromonas gingivalis.* ^37^

Three articles in this scoping review examined the use of *Limosilactobacillus reuteri* DSM 17938 as a probiotic intervention in periodontitis patients. Of these three studies, one included diabetic patients with periodontitis, while the other two used healthy individuals with no systemic disease. The probiotic was administered in two studies as a tablet containing a combination of *Limosilactobacillus reuteri* DSM 17938 and ATCC PTA 5289, while the third study used only *Limosilactobacillus reuteri* DSM 17938 in the form of a suspension delivered through a blunt syringe to subgingival sites. ^38^^–^^40^ Interestingly, the study with diabetic patients showed a significant reduction in the number of *Porphyromonas gingivalis*, *Tannerella forsythia*, and *Treponema denticola* after three weeks of probiotic tablet use, which was sustained after a three-month evaluation. ^38^ On the other hand, in line with the previous result, the study conducted by Vivekananda with a split-mouth study design in India found that only the treatment modalities that included the *Limosilactobacillus reuteri* tablet, either alone or in combination with scaling root planing, were able to significantly reduce the counts of pathogens tested ( *Aggregatibacter actinomycetemcomitans*, *Porphyromonas gingivalis*, and *Prevotella intermedia*). ^39^ Lastly, the study conducted by El-bagoory *et al.* (2021) showed a significant reduction in the number of *Porphyromonas gingivalis* on the three-month and six-month evaluations after only four applications of the probiotic suspension delivered to subgingival sites, even though it was administered only at baseline, week 1, week 2, and week 4. ^40^

The effectiveness of *Limosilactobacillus reuteri* in treating specific periodontal pathogens in peri-implant mucositis and periodontitis patients can vary depending on the distinct microbial network shape present in these niches. Peri-implant biofilm is more homogeneous and similar to supragingival biofilm than subgingival biofilm, while subgingival biofilm in periodontitis patients contains a higher number of microorganisms. In addition to this, the host response and habitat structure surrounding implants and teeth can also affect the microbial community structure and the effectiveness of probiotics. ^41^ Furthermore, the rough surface of dental implants increases the rate of biofilm formation around the implant, and surface roughness contributes to increased plaque buildup. ^42^

Based on our review, it was found that *Limosilactobacillus reuteri* is a probiotic bacterium that exhibits varying levels of antimicrobial activity against different pathogens. However, its effectiveness can be further enhanced by combining it with other clinical care and active ingredients such as natural compounds or prebiotics. For instance, Holm *et al.* (2022) showed that the antimicrobial effectiveness of *Limosilactobacillus reuteri* (ATCC PTA 5289) can be improved by combining it with glycerol against periodontal pathobionts and anaerobic commensals. This may be attributed to the increased production of reuterin by *Limosilactobacillus reuteri* from glycerol. ^43^ Hence, combining *Limosilactobacillus reuteri* with other active ingredients such as natural compounds or prebiotics may be a useful strategy for improving its antimicrobial efficacy. However, further details on this strategy need to be explored, including relevant references and future research perspectives. It is worth mentioning that some newly introduced compounds have a significant influence on the oral environment. The use of paraprobiotics, lysates, and postbiotics can modify clinical and microbiological parameters in periodontal patients. ^44^^–^^46^ Therefore, these products should be considered in combination with *Limosilactobacillus reuteri* in future clinical trials.

In conducting a scoping review, it is important to consider the limitations of the study design in order to interpret the findings in a meaningful way. One limitation of the current scoping review is the potential for bias in quality assessment due to the inclusion of a non-randomized study with a high risk of bias. However, the review also included 15 other studies, all of which were randomized control trials. Of these, 9 studies were found to have a low risk of bias, while 6 studies had “some concerns” regarding risk of bias based on the Cochrane Risk-Of-Bias (RoB 2) Tool. Although the non-randomized study may have impacted the overall quality of the evidence included in the review, the findings of the randomized control trials provide important insights into the effectiveness of the interventions studied. Moreover, the inclusion of grey literature in the search strategy can help to enhance the comprehensiveness of the review. Overall, while limitations should be acknowledged and considered in the interpretation of the findings, the scoping review still provides valuable insights and can serve as a foundation for future research in the field.

Furthermore, in this scoping review, it was found that several studies included in the analysis received materials and funding from a sponsor. Additionally, one author from a study received a PhD grant from the sponsor, while another author received a lecturing fee. Nonetheless, the authors of these studies stated that the involvement of the sponsor did not have any impact on the results of their research. It is essential to acknowledge the presence of external funding sources and their potential influence on study outcomes, as transparency in reporting is crucial in scientific research. Despite the presence of sponsorship, the authors of the reviewed studies made it clear that the research conducted was not compromised, and their findings were not influenced by the sponsors. However, it is necessary to approach the results with caution, as the presence of external funding sources can still potentially affect the outcome of research studies.

## Conclusions

*Limosilactobacillus reuteri* exhibits a strong antibacterial effect on *Streptococcus mutans* and has a less significant impact on *Lactobacilli* in healthy individuals. Additionally, *Limosilactobacillus reuteri* has antibacterial properties against *Porphyromonas gingivalis* in periodontitis and gingivitis patient although its effectiveness is not stable in patients with peri-implant infections. The study suggests that *Lactobacillus reuteri* does not have a noteworthy impact on *Campylobacter rectus* and *Eikenella corrodens.* However, the effectiveness of *Limosilactobacillus reuteri* varies concerning other bacteria such as *Prevotella intermedia, Peptostreptococcus micros, Aggregatibacter actinomycetemcomitans, Tannerella forsythia, Treponema denticola*, and *Fusobacterium nucleatum.* Further research is essential to examine the antibacterial properties of *Limosilactobacillus reuteri* on the oral/dental microbiomes, taking into account variations in the strains used, intervention periods, subjects, and observation times. This research is crucial in advancing our understanding of *Limosilactobacillus reuteri*’s potential as a probiotic for maintaining oral/dental health.

## Data Availability

No data are associated with this article. Harvard Dataverse: Search Strategy for the Article “The Antimicrobial Effect of Limosilactobacillus reuteri as Probiotic on Oral Bacteria: A scoping review”, https://doi.org/10.7910/DVN/IPHU9X. ^47^ Harvard Dataverse: PRISMA-ScR Checklist for the Article “The Antimicrobial Effect of Limosilactobacillus reuteri as Probiotic on Oral Bacteria: A scoping review”, https://doi.org/10.7910/DVN/EIGWI1. ^48^ Harvard Dataverse: PRISMA Flow Diagram for the Article “The Antimicrobial Effect of Limosilactobacillus reuteri as Probiotic on Oral Bacteria: A scoping review”, https://doi.org/10.7910/DVN/VJLVI9. ^49^ Data are available under the terms of the Creative Commons Zero “No rights reserved” data waiver (CC0 1.0 Public domain dedication).
