# Supplementary material for: The antimicrobial effect of  Limosilactobacillus reuteri as probiotic on oral bacteria: A scoping review
Source: F1000Res. 2024 Mar 4;12:1495. Originally published 2023 Nov 23. [Version 2] doi: 10.12688/f1000research.139697.2 (PMC10905032; doi:10.12688/f1000research.139697.2)
Supplement: Peer review history

# Reviewer report: Reviewer response for version 2

**DOI:** 10.5256/f1000research.163098.r252502

**Published:** 2024-03-27

**Authors:** Norifumi Nakamura

**Affiliations:** 1 Kagoshima University, Korimoto, Kagoshima, Japan

The revised version of this article has been improved following the reviewer's indications and suggestions.

Are the rationale for, and objectives of, the Systematic Review clearly stated?

Yes

Is the statistical analysis and its interpretation appropriate?

Yes

Are sufficient details of the methods and analysis provided to allow replication by others?

Yes

Are the conclusions drawn adequately supported by the results presented in the review?

Yes

Reviewer Expertise:

Oral and Maxillofacial Surgery, Reconstructive Surgery, Odontogenic Tumors

I confirm that I have read this submission and believe that I have an appropriate level of expertise to confirm that it is of an acceptable scientific standard.

# Reviewer report: Reviewer response for version 2

**DOI:** 10.5256/f1000research.163098.r252504

**Published:** 2024-03-14

**Authors:** Rizky Aditiya Irwandi

**Affiliations:** 1 University College London, London, UK

The authors have revised the manuscript accordingly and I am pleased to inform you that it is approved.

Are the rationale for, and objectives of, the Systematic Review clearly stated?

Yes

Is the statistical analysis and its interpretation appropriate?

Not applicable

Are sufficient details of the methods and analysis provided to allow replication by others?

Yes

Are the conclusions drawn adequately supported by the results presented in the review?

Yes

Reviewer Expertise:

oral microbiology and immunology

I confirm that I have read this submission and believe that I have an appropriate level of expertise to confirm that it is of an acceptable scientific standard.

# Reviewer report: Reviewer response for version 1

**DOI:** 10.5256/f1000research.152995.r237795

**Published:** 2024-02-09

**Authors:** Rizky Aditiya Irwandi

**Affiliations:** 1 University College London, London, UK

The authors provide a rather extensive review of the per-oral probiotics research, specifically its influence on oral microbiome not limited to oral-derived pathogens. Overall, the scoping review has adhered to the standardized criteria and all relevant information relevant to the review has been well delivered in detail. To achieve this, I would strongly recommend the authors to revise some aspects detailed below:

**Introduction**

 Paragraph 1: It would be concise to exclude the first paragraph.

 Paragraph 2: As the second paragraph becomes the first one, consider removing the word “Generally” would be appropriate.

 Paragraph 3: It would be concise to exclude the third paragraph.

 Paragraph 4: Consider removing “usually” from the first sentence and combining the first two sentences with conjunction words such as “and” would be more concise. For instance:

 “Oral microbiomes reside in the oral cavity as biofilms and maintaining their homeostasis is important to protect the oral cavity as the main gateway to the body. The shifted oral microbial balance may lead to diseases and antibiotics are a common antimicrobial approach to regain the oral microbiota equilibrium. Over-reliant antibiotic administration, however, increases the risk of developing antimicrobial resistance which WHO declared as one of the global health emergencies and is also known as a silent pandemic. Therefore, an alternative intervention such as probiotics gains attention to maintain oral biofilm homeostasis”

 Paragraph 5: removing “will” in the first sentence and “can” in the second and third as well as replacing “which is” with “by” in the second sentence and correcting the typo “pathogen” to “pathogenic” would help readers to better understand the ideas delivered by the authors.

**Results**

 The authors included RCT studies. I would recommend the authors include the RCT registration ID by clinicaltrial.gov or any other relevant RCT databases and present them in Table 1. This is particularly important to objectively assess each study confirming that indeed the RCTs designed by certain studies have fulfilled the criteria of the commonly used RCT databases.

 The authors mentioned that the review adheres to the PRISMA-ScR and that it would be easier for the reader to have a completed fillable checklist. This can be provided as supplemental information to the article. The authors may refer to this webpage for the checklist: http://www.prisma-statement.org/documents/PRISMA-ScR-Fillable-Checklist_11Sept2019.pdf

**Discussion**

 Paragraph one: the authors delivered ideas about the need for alternatives to antimicrobial approaches (in this case antibiotics) by harnessing bacterial quorum sensing. At the same time, however, the authors mentioned that quorum sensing leads to antibiotic resistance. I found this conflicting and this likely confuses the reader. I would recommend the authors revisit by restructuring this part.

 The use of active voices is, I would suggest, preferable to strengthen the clarity of ideas delivered. For example, the first sentences could be:

 “Oral biofilms consist primarily of commensal species that are in equilibrium and harmless to the host. However, both oral and systemic-derived insults are capable of inducing the alteration towards microbial dysbiosis leading to an increased number of pathogenic microbial species.”

 Minor errors:

 Paragraph 2: Replace “on the other side” with “moreover”

 Paragraph 3: Replace “conditions” with “disorders”

 The authors comprehensively discussed the studies included in the review. The detailed mechanisms of probiotic actions (for example, how probiotics are likely to influence bacterial quorum sensing) would provide a strong rationale for continuing the use of probiotics to regain the balance of oral microbiota. The ideas would be even more robust if the authors could expand more on the aspect such as to what extent the use of the probiotics is strongly recommended and perhaps any potential side effects should be highlighted (if no side effects are available, considering the explanation of the possible mechanism of this would be recommended).

Are the rationale for, and objectives of, the Systematic Review clearly stated?

Yes

Is the statistical analysis and its interpretation appropriate?

Not applicable

Are sufficient details of the methods and analysis provided to allow replication by others?

Yes

Are the conclusions drawn adequately supported by the results presented in the review?

Yes

Reviewer Expertise:

oral microbiology and immunology

I confirm that I have read this submission and believe that I have an appropriate level of expertise to confirm that it is of an acceptable scientific standard, however I have significant reservations, as outlined above.

# Reviewer report: Reviewer response for version 1

**DOI:** 10.5256/f1000research.152995.r226208

**Published:** 2024-02-07

**Authors:** Norifumi Nakamura

**Affiliations:** 1 Kagoshima University, Korimoto, Kagoshima, Japan

The antimicrobial activity of *Limosilactobacillus reuteri*, one of the probiotic strains, is of great interest and brings useful information to the field of dentistry

 However, the following major and minor problems are observed and should be corrected before acceptance.

 Major problems.

 1. It would be required to describe the results of the evaluation of the effect of the antibacterial activity of *Limosylactobacillus reuteri* on human oral bacteria based on the 16 papers that were finally selected.

 2. The discussion contains too many results and seems redundant. The discussion should be organized into important items with small headings.

 3. What are the criteria for arranging the 16 papers summarized in Table 1? Wouldn't it be easier for readers to read them if they were sorted by year of publication or evidence level of study design?

 Minor problems

 4. The article by Badari (2020), which was selected for Systematic review, is not in the list of references.

 5. The year in the text is different from the year in the list of references

 5. The text is well written, but is divided into too many small paragraphs.

Are the rationale for, and objectives of, the Systematic Review clearly stated?

Yes

Is the statistical analysis and its interpretation appropriate?

Yes

Are sufficient details of the methods and analysis provided to allow replication by others?

Yes

Are the conclusions drawn adequately supported by the results presented in the review?

Yes

Reviewer Expertise:

Oral and Maxillofacial Surgery, Reconstructive Surgery, Odontogenic Tumors

I confirm that I have read this submission and believe that I have an appropriate level of expertise to confirm that it is of an acceptable scientific standard.

# Reviewer report: Reviewer response for version 1

**DOI:** 10.5256/f1000research.152995.r226213

**Published:** 2023-12-19

**Authors:** Firdaus Hariri

**Affiliations:** 1 University of Malaya,, Kuala Lumpur,, Malaysia

Well done to the team of researchers for this valuable review.

 1) In general, the paper is well written and very structured.

 2) The objective and research question are clear and the methodology used to achieve the objective is comprehensively described supplemented with the application of appropriate review protocol.

 3) Results are well structured, supplemented with excellent summary tables.

 4) Discussion:

 - as *Limosilactobacillus reuteri* has been widely used as valuable probiotic in human, may I recommend a brief paragraph, either in 'Introduction' or at beginning of 'Discussion' on its usage in general health (authors only described its potential role in oral health).

 - to supplement the discussion on paragraph 12 (on diabetic patients) with studies about the role of *Limosilactobacillus reuteri *in diabetes mellitus (e.g metabolic or beneficial effect).

 Thank you.

Are the rationale for, and objectives of, the Systematic Review clearly stated?

Yes

Is the statistical analysis and its interpretation appropriate?

Not applicable

Are sufficient details of the methods and analysis provided to allow replication by others?

Yes

Are the conclusions drawn adequately supported by the results presented in the review?

Yes

Reviewer Expertise:

Metagenomic, oral health, oral and maxillofacial surgery

I confirm that I have read this submission and believe that I have an appropriate level of expertise to confirm that it is of an acceptable scientific standard.
